# Quantifying Isoprenoids in the Ergosterol Biosynthesis by Gas Chromatography–Mass Spectrometry

**DOI:** 10.3390/jof9070768

**Published:** 2023-07-20

**Authors:** Maximilian Liebl, Ludwig Huber, Hesham Elsaman, Petra Merschak, Johannes Wagener, Fabio Gsaller, Christoph Müller

**Affiliations:** 1 Department of Pharmacy, Center for Drug Research, Ludwig-Maximilians University, 81377 Munich, Germany; maximilian.liebl@cup.uni-muenchen.de (M.L.); lu.huber@campus.lmu.de (L.H.); 2 Institute for Hygiene and Microbiology, Julius-Maximilians-University Wuerzburg, 97080 Wuerzburg, Germany; hesham.elsaman@uni-wuerzburg.de (H.E.); wagenerj@tcd.ie (J.W.); 3 Institute of Molecular Biology, Biocenter, Medical University of Innsbruck, 6020 Innsbruck, Austria; petra.merschak@i-med.ac.at (P.M.); fabio.gsaller@i-med.ac.at (F.G.); 4 Department of Clinical Microbiology, School of Medicine, Trinity College Dublin, The University of Dublin, D08 RX0X Dublin, Ireland

**Keywords:** *Aspergillus fumigatus*, *erg1*, *erg9*, ergosterol biosynthesis, farnesol, GC-MS, isoprenoids, terbinafine

## Abstract

The ergosterol pathway is a promising target for the development of new antifungals since its enzymes are essential for fungal cell growth. Appropriate screening assays are therefore needed that allow the identification of potential inhibitors. We developed a whole-cell screening method, which can be used to identify compounds interacting with the enzymes of isoprenoid biosynthesis, an important part of the ergosterol biosynthesis pathway. The method was validated according to the EMEA guideline on bioanalytical method validation. *Aspergillus fumigatus* hyphae and *Saccharomyces cerevisiae* cells were lysed mechanically in an aqueous buffer optimized for the enzymatic deconjugation of isoprenoid pyrophosphates. The residual alcohols were extracted, silylated and analyzed by GC-MS. The obtained isoprenoid pattern provides an indication of the inhibited enzyme, due to the accumulation of specific substrates. By analyzing terbinafine-treated *A. fumigatus* and mutant strains containing tunable gene copies of *erg9* or *erg1*, respectively, the method was verified. Downregulation of *erg9* resulted in a high accumulation of intracellular farnesol as well as elevated levels of geranylgeraniol and isoprenol. The decreased expression of *erg1* as well as terbinafine treatment led to an increased squalene content. Additional analysis of growth medium revealed high farnesyl pyrophosphate levels extruded during *erg9* downregulation.

## 1. Introduction

Fungal infections threaten the health of more than one billion people worldwide. While most of them are inconvenient topical or vulvovaginal mycoses, some can be life-threatening invasive infections that lead to approximately 1.7 million deaths per year [1,2,3]. As a consequence of those often underrecognized emerging fungal threats that are accompanied by an increasing antimycotic resistance, the World Health Organization (WHO) recently published the first fungal priority pathogens list (October 2022) [4]. The document classifies pathogens in three priorities (critical, high and medium) by multicriteria decision analysis, considering *Aspergillus fumigatus*, *Candida albicans*, *Candida auris* and *Cryptococcus neoformans* as the most critical threats [3,4]. An accelerator increasing the number of invasive mycoses such as mucormycosis, aspergillosis and candidiasis was the global coronavirus disease 2019 (COVID-19) [5,6,7]. Patients suffering from severe acute respiratory syndrome coronavirus type 2 (SARS-CoV-2) have a significantly lower number of T-cells; in addition, the first-line treatment of severe COVID-19 pneumonia with corticosteroids impairs the immune system [6,8]. In an aggressive disease course, SARS-CoV-2 may even damage lunge tissue and cause alveolar-intestinal lesions, leading to a higher susceptibility for pulmonary infections [8].

Considering those current hazards caused by fungal germs, potent antifungal therapies are mandatory. The first time a targeted antifungal therapy became available was in the 1950s with the introduction of the polyene amphotericin B (AMB), a broad-spectrum antifungal agent that is still the first-line treatment of invasive mycoses [1]. Besides polyenes that can interact with ergosterol directly, several clinically relevant classes of ergosterol biosynthesis inhibitors including imidazoles/triazoles (e.g., clotrimazole/fluconazole), morpholines (e.g., amorolfine) and allylamines (e.g., terbinafine) were launched on the market. The efficacy of those sterol biosynthesis inhibitors is based on the pivotal role of ergosterol for fungal membrane flexibility, rigidity and permeability. Even though the efficacy of imidazoles/triazoles is indisputable, all known ergosterol biosynthesis inhibitors exclusively inhibit enzymes of the post-squalene pathway [9], whereas the pre-squalene part was disregarded until now.

**Figure 1:**
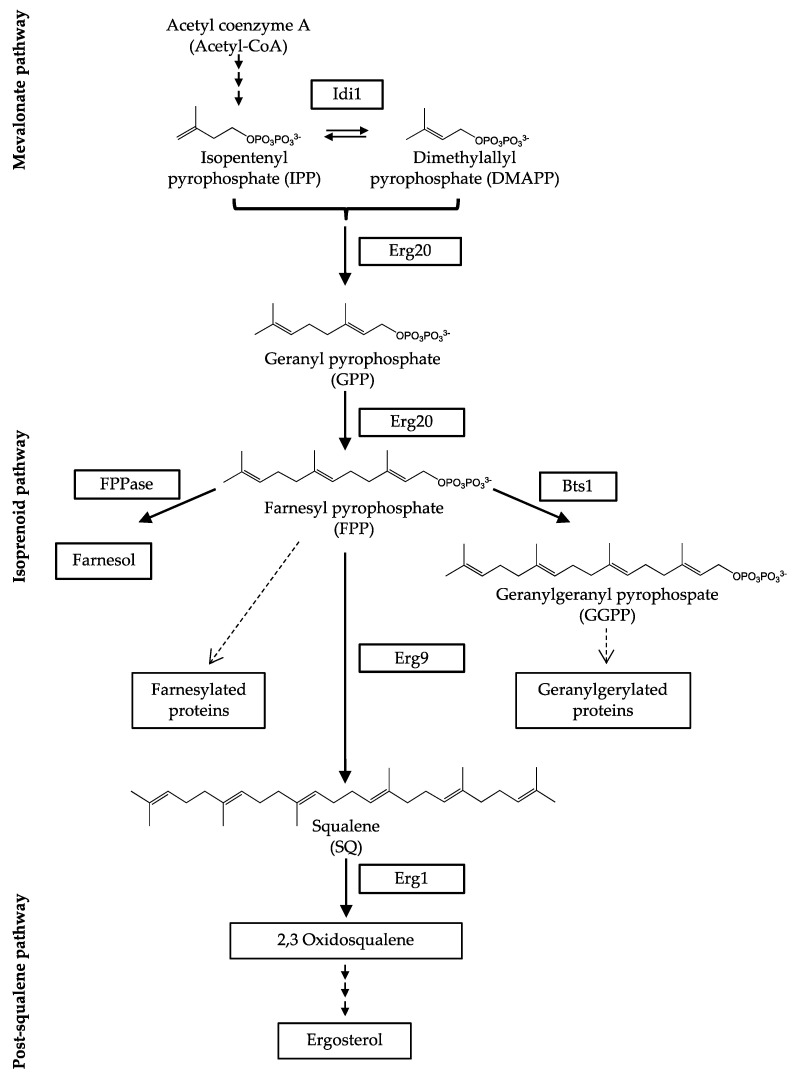
Scheme of ergosterol biosynthesis in fungi with the isoprenoid pathway in detail. Farnesyl pyrophosphatase (FPPase); farnesyl pyrophosphate synthase (Erg20); geranylgeranyl pyrophosphate synthase (Bts1); isopentenyl pyrophosphate isomerase (Idi1); squalene epoxidase (Erg1), squalene synthase (Erg9).

Isoprenoids with the formula (C_5_H_8_)_n_ are formed from acetyl-coenzyme A (acetyl-CoA) in the mevalonate pathway, the first part of the pre-squalene pathway. From here on, isoprenoids are involved in the synthesis of numerous bioactive compounds. Besides the biosynthesis of sterols, they take part in the formation of dolichols, ubiquinone, as well as the post-translational modifications of proteins by prenylation reactions (Figure 1) [10,11].

Dimethylallyl pyrophosphate (DMAPP) and isopentenyl pyrophosphate (IPP) are the substrates for the biosynthesis of terpenes. Both molecules are hemiterpenes (C_5_H_8_) that can be interconnected by the enzyme isopentenyl pyrophosphate isomerase (Idi1) [11,12,13]. In a head-to-tail condensation, each equivalent DMAPP and IPP result in the monoterpene (C_10_H_16_) geranyl pyrophosphate (GPP) [12,14]. Therefore, the reaction is catalyzed by the enzyme farnesyl pyrophosphate synthase (Erg20), which also catalyzes the following reaction from GPP to farnesyl pyrophosphate (FPP) using a second unit of IPP [14]. With the sesquiterpene (C_15_H_24_) FPP, the reaction cascade reaches a branching point, where FPP can be directly converted to farnesol by farnesyl pyrophosphatase (FPPase), or used in other biosynthesis reactions outside sterol biosynthesis, e.g., biosynthesis of carotenoids, sesquiterpenoids and *N*-glycans [15,16,17,18]. The diterpene (C_20_H_32_) geranylgeranyl pyrophosphate (GGPP) is synthesized from FPP and IPP in an addition-elimination-condensation catalyzed by geranylgeranyl pyrophosphate synthase (Bts1) [16]. GGPP is not a precursor of ergosterol but is also used for the biosynthesis of further bioactive molecules, e.g., G-proteins, ubiquinone and carotenoids [19,20]. The major part of FPP is used for the synthesis of the triterpene (C_30_H_50_) squalene (SQ), which is the final product of the isoprenoid pathway [11,15,16,21]. Squalene is synthesized by squalene synthase (Erg9) starting from two units of FPP. In contrast to the post-squalene/lanosterol pathway, which contains specific intermediates depending on the organism, the isoprenoid pathway intermediates are identical in fungi, mammals and plants [21]. Also, in several bacteria, e.g., *Escherichia coli* or *Staphylococcus aureus*, isoprenoids can be detected [22,23].

For the development of novel antifungals, the isoprenoid part of the pre-squalene pathway could be a promising target since its enzymes play an essential role in fungal cell growth [13,15,24,25,26,27]. Those findings were confirmed by studying the impact of conditional downregulation of *erg9* and *erg1* on the growth of *A. fumigatus* (Supplementary Material Figure S1). In the literature, evidence can be found that also a deletion of *erg20* results in a lack of FPP and is lethal to fungal cells, due to the necessity of FPP as a metabolic intermediate. In addition, an accumulation of monoterpenes can be toxic for microorganisms [26,27]. The essential role of *IDI1* in *S. cerevisiae* was highlighted by Mayer et al. [13].

Even though the precursors of ergosterol and involved enzymes are well known, their qualitative and quantitative measurement remains difficult due to their widely varying physicochemical properties. Molecules of the post-squalene pathway are lipophilic because of their sterol backbone with one or two hydroxyl groups, which makes them accessible for analysis by gas chromatography (GC) [9,28,29]. In contrast, the compounds of the pre-squalene pathway consist of phosphorylated isoprenoids that are negatively charged under physiological conditions. The phosphorylated isoprenoids are better accessible for the analysis by liquid chromatography (LC) due to their water solubility. To make isoprenoids vaporable and available for a GC analysis, the isoprenoids must be dephosphorylated. Besides acidic or basic hydrolysis, enzymatic deconjugation is a convenient way to generate isoprenoids [30,31,32,33]. Nevertheless, only few approaches exist where the intermediates of the pre-squalene pathway are analyzed. An overview of methods described in the literature for the analysis of intermediates of the pre-squalene pathway is given in Table 1. The first assays describing the analysis of intermediates of the pre-squalene pathway were using radioactive labeled precursors such as [5-^3^H]mevalonolactone [34], [1-^14^C]isopentenyl diphosphate or [1-^3^H]farnesyl diphosphate [35].

For the analysis of these radioactively labeled analytes, a scintillation counter was used. In the early 2000s, an increasing number of cellular models was established where radioactive labeling methods were replaced with mass spectrometry (MS) detection, a radioactivity-free detection method. This has led to the development of several efficient high-performance liquid chromatography (HPLC) and GC-MS methods that were limited to single analytes and did not cover the whole isoprenoid pathway. Henneman et al. [39] published an assay in 2008 that is based on human Hep2G cells, covering eight analytes of the pre-squalene pathway including mevalonate and its mono- and di-phosphate species, intermediates of the mevalonate pathway. Due to the use of an HPLC–MS/MS system, the analytes do not have to be dephosphorylated and can be analyzed directly. However, a distinction between the isomers IPP and DMAPP was not possible with this approach [39]. An effective GC-MS method was applied by Huang et al. [33] in 2011. In their approach, they were able to quantify four intermediates of the isoprenoid pathway including geraniol (GOH), farnesol (FOH), geranylgeraniol (GGOH) and squalene (SQ). However, the method was not very efficient, considering incubation periods (105 min for the dephosphorylation step), the total run time of the GC method (37 min) and the necessity of large amounts of biomass (100 mL of an OD_600_~0.1; incubation for 48 h at 30 °C). In addition, method validation was not performed in line with the appropriate analytical guideline, resulting in missing thresholds for the validation parameters [33]. Rodriguez et al. [17] were capable of analyzing mevalonate (MVA) in addition to FOH and SQ levels from fungal cells. Therefore, three different GC-MS methods had to be used to cover the whole scope of analytes: metabolite profiling of MVA, metabolic profiling of sesquiterpenes including FOH, and metabolic profiling of SQ and ergosterol. Chhonker et al. [40] analyzed isoprenoids from human cancer cell lines and plasma using HPLC–MS/MS. Even though they were able to quantify the intermediates GPP, FPP and GGPP at basal levels, their protocol did not cover the remaining isoprenoid biosynthesis pathway, missing IPP, DMAPP and SQ. In a more recent work, Castaño-Cerezo et al. [41] used an HPLC-high-resolution (HR)MS system to quantify the eight intermediates of the pre-squalene pathway in *S. cerevisiae*. Compared to Henneman et al. [39], they were able to implement several improvements; however, the limitations of the LC system remain. Therefore, the separation and distribution of IPP and DMAPP was not possible in their approach. 

In this work, we present a novel, selective and reproducible assay to analyze the intermediates of the isoprenoid pathway, including the isomers IPP and DMAPP, in fungal cell matrices (*A. fumigatus*, *S. cerevisiae*), using a quick and simple approach. The developed methodology was validated according to European Medicines Agency (EMEA) guideline on bioanalytical method validation [42]. As proof of concept, we analyzed lyophilized *A. fumigatus* cells and their associated growth medium as well as terbinafine-treated wild type (wt) cells to give detailed insights into isoprenoid and sterol trafficking in fungal cells for the first time. 

## 2. Materials and Methods

### 2.1. Chemicals

**Solvents:** All solvents were purchased in HPLC grade or in *pro analysis* quality from Sigma Aldrich (Schnelldorf, Germany). Deionized water was prepared with an in-house ion-exchanger. Double-distilled water was purchased from Fresenius Kabi Deutschland GmbH (Bad Homburg, Deutschland).

**Standards:** Dimethylallyl pyrophosphate (DMAPP) (95%), farnesyl pyrophosphate (FPP) (95%) and geranylgeranyl pyrophosphate (GGPP) (95%) were from Cayman Chemicals (Michigan, MI, USA); prenol (POH) (97%) and farnesol (FOH) (97%) were purchased from Alfa Aesar (Karlsruhe, Germany). Isoprenol (IOH) (98%) was from Tokyo Chemical Industry Co. (Tokyo, Japan). Geraniol (GOH) (98%) and squalene (SQ) (98%) were purchased from Sigma Aldrich (Steinheim, Germany); geranylgeraniol (GGOH) (85%) and 1–heptadecanol (internal standard for isoprenoid analysis, IS_Iso_) (98%) were from Sigma Aldrich (Saint Louis, MO, USA). The second internal standard for sterol analysis, 5α-cholestane (internal standard for sterol analysis, IS_Sterol_) (97%), was purchased from Sigma Aldrich (Steinheim, Germany). Doxycycline monohydrate (97%) was purchased from Sigma Aldrich (Steinheim, Germany) and terbinafine (98%) was obtained from BLDpharm (Kaiserslautern, Germany).

**Enzymatic deconjugation step:** Bovine alkaline phosphatase (P7640) was purchased from Sigma Aldrich (Steinheim, Germany). Diethanolamine (99%) was purchased from Tokyo Chemical Industry Co. (Tokyo, Japan). Magnesium chloride hexahydrate (99%) and glycine (98%) were purchased from Sigma Aldrich (Steinheim, Germany). Sodium chloride was purchased from Bernd Kraft GmbH (Duisburg, Germany).

**Derivatization step:** *tert*-Butyldiphenylchlorosilane (*t*BDPSCl) (98%) and imidazole (99%) from Sigma Aldrich (Steinheim, Germany) were used for derivatization of isoprenoids. For silylation of sterols, a mixture of *N*-methyl-*N*-trimethylsilyltrifluoroacetamide (MSTFA) and *N*-trimethylsilylimidazole (TSIM) from Macherey Nagel (Düren, Germany) was used (10/1; *v*/*v*). 

### 2.2. Reagents

Analyte and internal standard (IS_Iso_, 1–heptadecanol; IS_Sterol,_ 5α-cholestane) stock solutions (each 1 mg/mL) were prepared in *n*-hexane. Working standard solutions were prepared (100 µg/mL in *n*-hexane) and diluted as needed. From pyrophosphates (DMAPP, FPP, GGPP), equimolar working solutions corresponding to the analyte concentration in µg/mL were prepared in methanol/ammonium hydroxide (7/3; *v/v*). IS_Iso_ and IS_Sterol_ stock solutions were diluted and combined to achieve one internal standard working solution containing 50 µg/mL (IS_Iso_) and 10 µg/mL (IS_Sterol_). All stock solutions were stored at −20 °C, tempered for 1 h and shaken before use. The aqueous 1 M diethanolamine buffer solution pH 8.6 was prepared in a stock of 100 mL containing magnesium chloride hexahydrate (0.5 mM) (DEA-buffer). Working solutions and buffers were stored at 4 °C, tempered for 15 min and shaken before use.

### 2.3. Fungal Strains Used in This Study

The non-homologous end-joining-deficient *A. fumigatus* strain AfS35, a derivative of D141, served as the wt strain in this study [43,44]. The conditional *erg1_tetOn_* and *erg9_tetOn_* strains were generated by replacing the promoters of the respective genes (*erg1*, AFUA_5G07780; *erg9*, AFUA_7G01220) with a doxycycline-inducible Tet-On promoter, essentially as described before [45,46]. *S. cerevisiae* was obtained from DSMZ (German Collection of Microorganisms and Cell Cultures; DSM-No. 1333, Braunschweig, Germany). 

### 2.4. Fungal Growth and Culture Conditions

For sterol analysis, wt and tunable *A. fumigatus* strains were grown in *Aspergillus* minimal medium (AMM) containing 20 mM ammonium tartrate as the nitrogen source and 1% glucose as the carbon source [47]. Cultures were inoculated with a final concentration of 1.0 × 10^6^ spores/mL of each strain and incubated for 20 h at 37 °C, 200 rpm. Mycelia were harvested through filtration, shock-frozen and freeze-dried. 

For *S. cerevisiae*, the DSMZ 186 universal medium for yeast was used for cultivation. The medium contained 3.0 g of yeast extract, 10.0 g of glucose and 15.0 g of agar from Sigma Aldrich (Steinheim, Germany); 3.0 g of malt extract from AppliChem GmbH (Darmstadt, Germany); and 5.0 g of peptone from soyabean from Oxoid (Basingstoke, Hampshire, UK). All components were dissolved in 1000 mL of deionized water and autoclaved for 10 min at 121 °C before use. Cell cultures were maintained at 28 °C and split once a week to keep the yeast in a log phase [28,48].

Cell matrix for method development and validation was obtained by incubating 2 mL of 5.0 × 10^5^ CFU/mL in DSMZ 186 medium in 24-well plates at 28 °C for 48 ± 2 h. 

### 2.5. Instruments and Equipment

#### 2.5.1. Sample Preparation

For shaking, a Vortex-Genie 2 by Scientific Industries, Inc. (Bohemia, NY, USA) was used. Centrifugation of 2 mL microcentrifuge tubes was performed with an Eppendorf 5415 D centrifuge (Hamburg, Germany). Derivatization and enzymatic deconjugation were conducted in a Binder ED23 laboratory drying cabinet from VWR (Ismaning, Germany).

#### 2.5.2. GC-MS Analysis of Isoprenoid tBDPS Ethers, Squalene and Sterol TMS Ethers

Gas chromatography was performed on an Agilent 7820A gas chromatograph coupled to a quadrupole 5977B MS from Agilent (Santa Clara, CA, USA). The 7693A automatic liquid sampler (ALS) from Agilent (Santa Clara, CA, USA) was used with the G4513A split/splitless injector from Agilent (Santa Clara, CA, USA). Data analysis and instrument control were carried out with the Masshunter 8.0 software from Agilent (Santa Clara, CA, USA). The column was an Agilent DB5-ms capillary column (Santa Clara, CA, USA) of 30 m in length, 0.25 mm in inner diameter and 0.25 µm in film thickness. Chromatography was performed with 99.9990% ALPHAGAZ^TM^ 1 HELIUM/He from Air Liquide (Düsseldorf, Germany) as the carrier gas at a constant flow rate of 1.4 mL/min. The inlet temperature was kept at 270 °C and the injection volume was 1 µL. Initial column temperature was 75 °C, which was held for 0.5 min. With a heating rate of 25 °C/min, temperature was increased to 180 °C until it was held for 1.0 min. Subsequently, temperature increased to 225 °C with a heat rate of 15 °C/min. After reaching 225 °C, the heating rate was increased to 50 °C/min to reach the final temperature of 320 °C, where the column was held for 5.9 min. The total run time was 16.5 min, followed by a 2.5 min post run with an increased flow rate of 2.0 mL/min. Transfer line temperature was 270 °C. The ion source temperature was 230 °C and the quadrupole temperature was 150 °C. The detection of the isoprenoid *t*BDPS ethers and squalene started after a solvent delay of 9.5 min in single-ion-monitoring (SIM) mode (see details in Table 2) at 70 eV. A selected ion chromatogram of the quantifier ions from different concentrations can be seen in Supplementary Material Figure S2. In addition, SIM and full-scan spectra (50–500 (*m*/*z*)) of the isoprenoid ethers, IS_Iso_ and squalene can be seen in Supplementary Material Figures S3 and S4, respectively.

Analysis of sterol TMS ethers was performed according to Müller et al. [9,28]. The sterol TMS ethers were analyzed in full scan mode by GC-MS (Table 3). The amount of each sterol is expressed as µg per mg dry weight. The results were obtained with a method that was not fully validated for the extraction solvent *n*-hexane. 

#### 2.5.3. Final Sample Preparation

**Figure 2:**
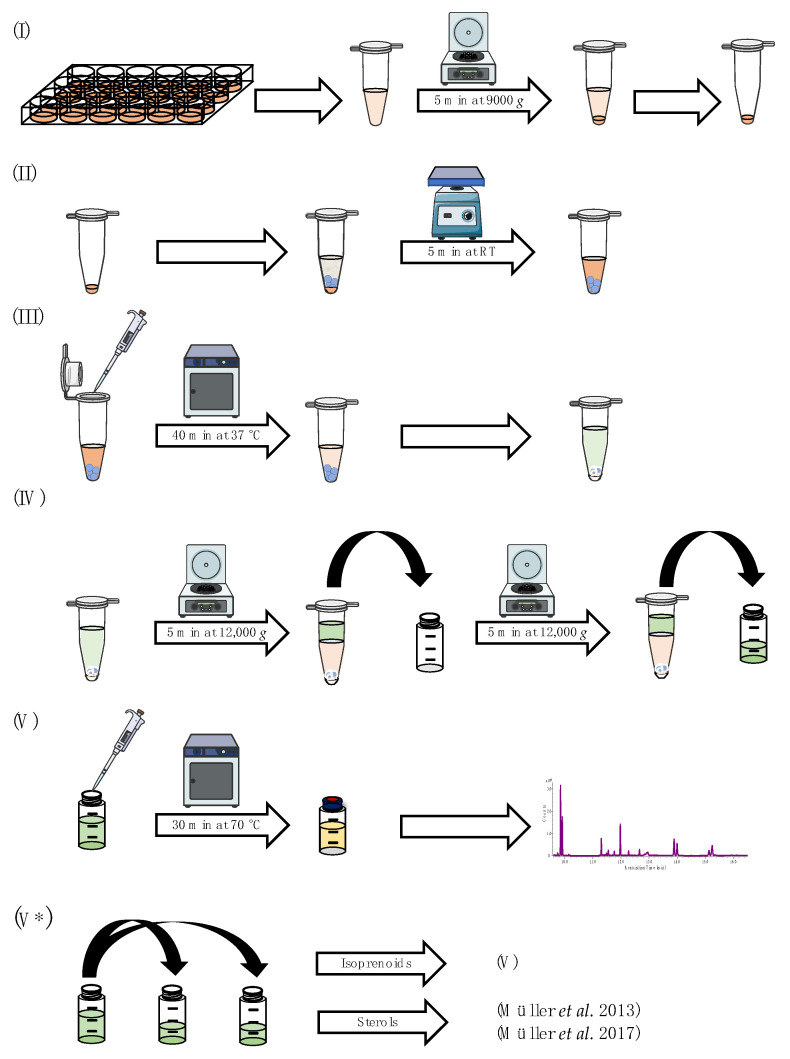
Schematic overview of the sample preparation, which can be divided into five individual steps: (**I**) preparation of cellular matrix; (**II**) mechanical lysis of cells/lyophilized mycelia in enzymatic buffer; (**III**) enzymatic deconjugation and preparation of liquid–liquid extraction; (**IV**) liquid–liquid extraction; (**V**) derivatization and GC-MS measurement; (V*) alternative sample preparation for additional sterol pattern analysis [9,28]. Parts of this figure were created using Servier Medical Art templates, licensed under a Creative Commons Attribution 3.0 Unported License (https://smart.servier.com (accessed on 19 July 2023).

For the analysis of *S. cerevisiae* samples, the cell suspension of each well was transferred into a 2.0 mL microcentrifuge safe-lock tube and centrifuged for 5 min at 9000× *g* at room temperature (RT). After centrifugation, the supernatant was decanted from the cell pellet (Figure 2I). For the analysis of *A. fumigatus* samples, lyophilized mycelia were pulverized, and 5 ± 0.2 mg was transferred into a 2.0 mL microcentrifuge safe-lock tube.

**Figure 3:**
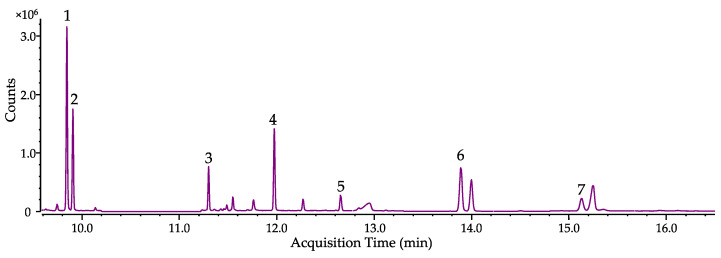
Selected ion chromatogram of spiked isoprenes (1000 ng/mL) detected as *t*BDPS ethers (squalene is not derivatized) in *A. fumigatus*. Isoprenol (1), prenol (2), geraniol (3), squalene (4), farnesol (5), IS_Iso_ (6), geranylgeraniol (7).

For mechanical cell lysis, three 1.5 mm and three 3.0 mm glass beads were added to the remaining cell pellet/lyophilized cells and resuspended in 590 µL of DEA-buffer, before the microcentrifuge safe-lock tube was vortexed for 5 min (Figure 2II). Then, 10 µL of a bovine alkaline phosphatase suspension in DEA-buffer (0.4 mg/mL) was added and the mixture was incubated for 40 min at 37 °C. Afterward, the enzymatic reaction was stopped by adding 300 ± 6 mg of NaCl, 300 µL of acetonitrile/acetone (2/1; *v*/*v*), 350 µL of *n*-hexane and 100 µL of the internal standard mixture containing 1–heptadecanol (IS_Iso_, 50 µg/mL in *n*-hexane) and cholestane (IS_Sterol_, 10 µg/mL in *n*-hexane) (Figure 2III). After vigorously shaking for 1 min per hand, the sample was centrifuged for 5 min at 12,000 × *g* at RT. Then, 450 µL of the upper *n*-hexane layer was transferred into a GC-vial (first extraction step). The mixture was extracted a second time in the same manner with another 750 µL of *n*–hexane (second extraction step). After centrifugation, 650 µL of the organic upper layer was transferred to the GC-vial from the first extraction step (Figure 2IV). The combined organic extracts were derivatized by adding 30 µL of *tert*-butyldiphenylchlorosilane and 30 µL of imidazole solution (262 mg/mL in tetrahydrofuran). For complete derivatization, the sample was stored for 30 min at 70 °C before being subjected to GC-MS analysis (Figure 2V). Analysis of culture supernatant harvested after incubation was conducted using the residues from 1.0 mL of freeze-dried medium and resuspending it in 590 µL of DEA-buffer (starting at Figure 2II). A chromatogram of spiked *A. fumigatus* sample is shown in Figure 3.

For additional sterol analysis, the combined organic extracts were split. Five hundred microliters was transferred into a new GC-vial and evaporated to dryness before the sample was analyzed according to Müller et al. [9,28]. For the analysis of the isoprenoids, the remaining 600 µL of the organic extract was derivatized as described above (Figure 2V*).

### 2.6. Method Validation

Method validation was performed according to the EMEA guideline on bioanalytical method validation EMEA/CHMP/EWP/192217/2009 [42]. According to the guideline, the following criteria were determined: selectivity, linearity, lower limit of quantification (LLOQ), accuracy, precision, carry over, dilution integrity, matrix effects and stability. In addition, the parameters recovery and injection precision were determined (Supplementary Material, Method validation). 

### 2.7. Growth Tests of the Conditional erg9_tetOn_ and erg1_tetOn_ Strains

A total of 1500 conidia suspended in 3 µL of double-distilled water were spotted on AMM plates and incubated at 37 °C for 48 ± 2 h. The plates were then photographed and the diameter of each colony was determined using ImageJ [49]. The strains were tested in triplicates.

### 2.8. Identification of Isoprenoid Patterns in A. fumigatus

The applicability of the previously validated method was tested on lyophilized cells and lyophilized growth medium from *A. fumigatus.* Cells from doxycycline inducible mutant strains were compared to doxycycline-treated wt cells. In addition, a distinction between intermediates originating from pyrophosphates that were generated by enzymatic deconjugation during sample preparation and their corresponding free alcohols was made. This was accomplished by splitting one lyophilized sample into two samples. The samples were analyzed with/without enzymatic deconjugation step (see Section 2.5.3, Figure 2III). Besides mutant strains that contain inducible gene variants of *erg9* or *erg1*, terbinafine-treated wt cells were also analyzed and compared to untreated wt cells.

## 3. Results

### 3.1. Sample Preparation

#### 3.1.1. Extraction Solvent

**Figure 4:**
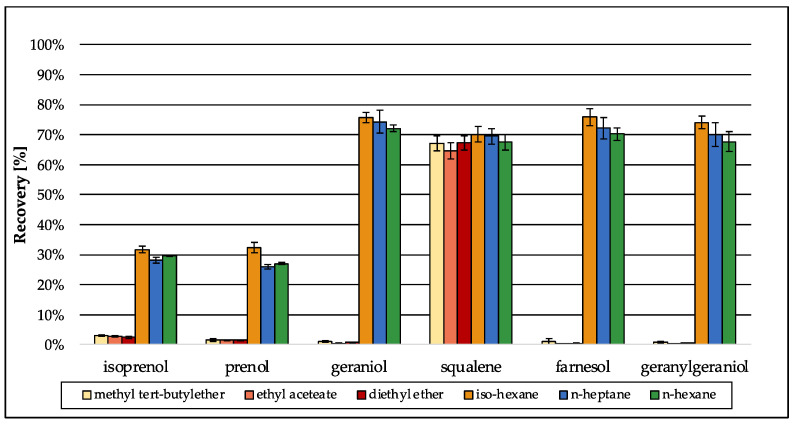
Preliminary recovery of the analytes during method development in different organic solvents that were tested for their capability to extract isoprenoids from water. Values were normalized using standards of the analytes (1000 ng/mL) in *n*-hexane. Error bars represent the standard deviation of five technical replicates.

Several methods have been described for the extraction of lipids and sterols from biological matrices, mainly based on the well-known methods from Bligh and Dyer [50] or Folch et al. [51]. Both original methods use the cancerogenic organic solvent chloroform in combination with methanol, which were replaced by ethyl acetate and ethanol in more recent approaches [52]. For the extraction of sterols, more specific methods have been developed, performing liquid–liquid extraction using only one organic solvent, e.g., diethyl ether, *n*-hexane or methyl *tert*-butyl ether [9,53]. Due to the aim of simultaneous analysis of the intermediates of the isoprenoid pathway along with distal sterol biosynthesis intermediates, method development was based on an approach for sterol extraction [9]. Preliminary tests using water revealed that *n*-hexane is the best organic solvent for extraction of isoprenoids (Figure 4, green bars: *n*-hexane). Even though *iso*-hexane and *n*-heptane were as efficient as *n*–hexane in terms of recovery, their recovery values were accompanied by a higher standard deviation (SD). Because recovery should be further improved during method development, a low SD was preferred in the beginning of method development. Especially for the small terpenes isoprenol and prenol that were extracted most inefficiently, the SD was low when *n*-hexane was used. Diethyl ether, ethyl acetate and methyl *tert*-butyl ether were not capable of extracting the isoprenoids in an efficient way compared to *iso*-hexane, *n*-heptane or *n*-hexane.

**Figure 5:**
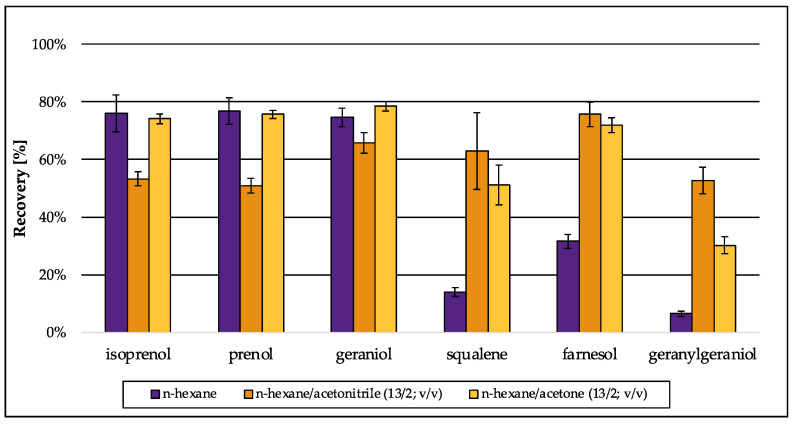
Preliminary recovery of isoprenoids extracted from *S. cerevisiae* matrix during method development: *n*-hexane was used as single extraction solvent or in combination with acetonitrile or acetone. Values were normalized using standards of the analytes (1000 ng/mL) in *n*-hexane. Error bars represent the standard deviation of five technical replicates.

The extraction procedure was improved by salting out the aqueous phase using sodium chloride in excess, to increase the recovery of the short-carbon-chain alcohols isoprenol and prenol (C_5_) that are better miscible with water than the C_10_–C_20_ isoprenoids. In matrix-matched approaches, (*S. cerevisiae*) recovery for all analytes decreased due to interactions of the isoprenoids with the cellular matrix (Figure 5, purple bars: *n*-hexane). To increase recovery in matrix-matched approaches again, mixtures of *n*–hexane with acetonitrile or acetone in the first of two extraction steps were efficient. Especially, farnesol (FOH) and geranylgeraniol (GGOH) were extracted more successfully when only one of those organic solvents was present, whereas the extractability for the smaller hemiterpenes (C_5_) decreased in the presence of acetonitrile (Figure 5). 

The addition of acetonitrile (first extraction: *n*-hexane/acetonitrile (11/4; *v*/*v*)) was most beneficial for the extraction of larger terpenes (squalene, farnesol, geranylgeraniol), whereas the extraction for the smaller terpenes (isoprenol, prenol, geraniol) with acetonitrile (first extraction: *n*-hexane/acetonitrile (11/4; *v*/*v*)) was less effective. The use of *n*-hexane/acetone (first extraction: *n*-hexane/acetone (11/4; *v*/*v*)) gave similar results for the smaller terpenes (isoprenol, prenol, geraniol) as pure *n*-hexane, but lower recovery levels especially for geranylgeraniol (Figure 5). Therefore, the optimized extraction solvent was a mixture of *n*-hexane, acetone and acetonitrile (9/4/2; *v*/*v*/*v*) in the first extraction step. Without the addition of the solvent mixture in the first extraction step, recovery of geranylgeraniol was below 10% in matrix-matched samples, which could be improved to >40% (Table 4). Hence, the final solvent mixture for the salt-assisted liquid–liquid extraction (SALLE) was a mixture of *n*-hexane/acetone/acetonitrile (9/4/2; *v*/*v*/*v*) for the first extraction step followed by a second extraction with *n*-hexane (ratio after second extraction step: *n*-hexane/acetone/acetonitrile (12/2/1; *v*/*v*/*v*) (see Supplementary Material, S2.7 Recovery).

#### 3.1.2. Enzymatic Deconjugation

**Figure 6:**
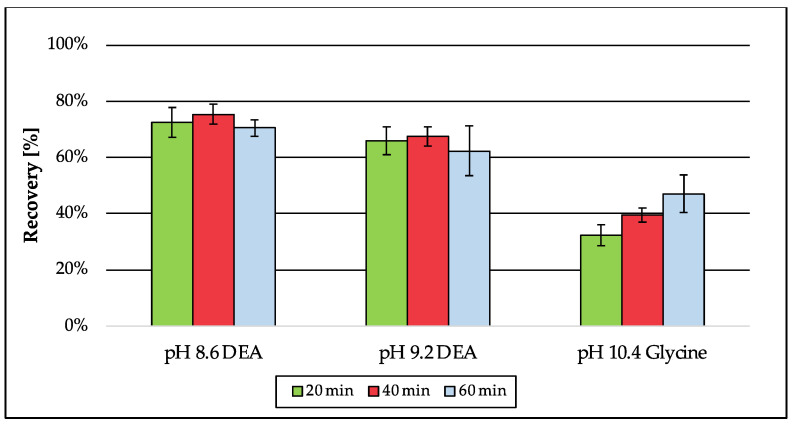
Recovery of prenol generated by enzymatic deconjugation from dimethylallyl pyrophosphate depending on pH and incubation time. The samples were dissolved in the corresponding buffers and normalized using standards of prenol in *n*-hexane (5 µM). Error bars represent the standard deviation of six technical replicates.

**Figure 7:**
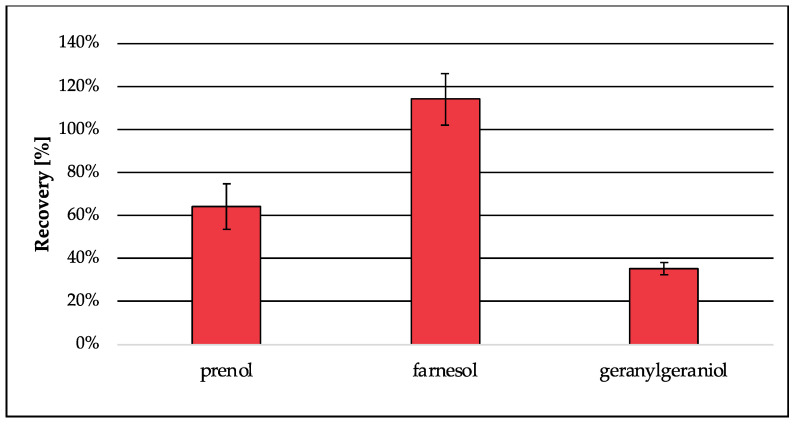
Recovery of prenol, farnesol and geranylgeraniol generated by enzymatic deconjugation from their corresponding pyrophosphates in matrix (*S. cerevisiae*). Peak areas were normalized using matrix-matched standards of the analytes (5 µM). Error bars represent the standard deviation of six technical replicates.

For the analysis of isoprenoids as *t*BDPS ethers by GC-MS, the free alcohols of the pyrophosphorylated isoprenoids are mandatory. For this reason, an additional step for deconjugation is essential. According to literature, enzymatic deconjugation is a convenient way to perform ester cleavage of phosphates and pyrophosphates. Therefore, the use of phosphatase is an established method [30,31,32,33]. The enzyme is most commonly obtained from bovine intestine [31,32] but can also be extracted from human placenta [30], bacteria or fungi [33]. Due to the different sources the enzyme can originate from, specific activity optima depending on the buffer system, pH, temperature and incubation time had to be considered. In contrast to Huang et al. [33], where two specific enzymes were needed (pyrophosphatase and phosphatase) to cleave the diphosphate esters, in this approach, only bovine alkaline phosphatase was used, which is capable of performing as a diphosphatase as well as an orthophosphatase. The use of a diethanolamine (DEA) buffer according to the manufacturer’s protocol generates a higher enzyme activity for alkaline phosphatase from bovine kidney compared to tris or glycine buffers [54]. The buffer was supplemented with magnesium chloride, which is essential for the orthophosphatase activity but can also be rate-limiting for diphosphatase activity, if concentrations were not adjusted [31]. As a critical parameter, the pH was adjusted to 8.6, whereas higher pH values were accompanied by decreased recovery (Figure 6). Recovery for the analyte dimethylallyl pyrophosphate was best when the enzymatic reaction was stopped after 40 min compared to shorter and longer incubation times. Also, the amount of enzyme was considered, because the ability of enzymes to catalyze reactions is not limited to one direction. The optimized enzymatic deconjugation step consists of 40 min of incubation time at 37 °C using a 1 M diethanolamine buffer pH 8.6 containing magnesium chloride hexahydrate (0.5 mM). In a matrix-matched approach (Figure 7), the recovery of prenol (64%) was slightly decreased compared to the previous tests in buffer (75%) (Figure 6), whereas the extraction of farnesol was 114% and was therefore quantitative. The recovery of geranylgeraniol was 35%, which could already be expected due to the lower recovery from extraction (see Section 3.1.1; Table 4).

### 3.2. Validation of the Test System

An overview of tested parameters and the results is given in Table 5. For full validation data and more detailed explanation, see Supplementary Material: S1. Method validation and S2. Validation results.

### 3.3. Application on Biological Samples from A. fumigatus

#### 3.3.1. Analysis of the Conditional *erg9* Strain

**Figure 8:**
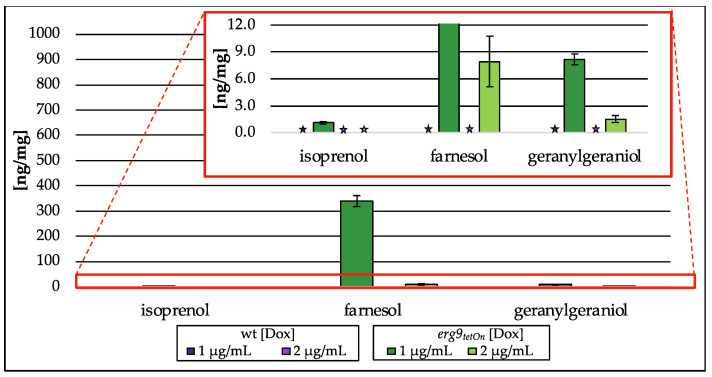
Amount of isoprenol, farnesol and geranylgeraniol in ng per mg biomass (dry weight) extracted from the conditional *erg9_tetOn_* strain cultured with different concentrations of doxycycline (Dox; 1 µg/mL, 2 µg/mL). In the red box, the zoomed *y*-axis is depicted. Error bars represent the standard deviation from three biological replicates, each analyzed as technical duplicates. ★ not detected (n.d.).

Downregulation of *erg9* gene expression leads to the inability of *A. fumigatus* to grow (Supplementary Material Figure S1). In the presence of low doxycycline concentrations (1 µg/mL and 2 µg/mL), growth of the conditional *erg9* strain is still possible, although reduced when compared to the wt or the fully induced *erg9* mutant strain. As shown in Figure 8, this reduced growth is accompanied by an accumulation of the free alcohols isoprenol, farnesol and geranylgeraniol (Figure 8). This accumulation of free alcohols, corresponding to their isoprenoid pathway intermediates, is Dox-dependent and reaches from n.d. (2 µg/mL Dox) to approx. 1 ng/mg (on dry weight basis (dw); 1 µg/mL Dox) of isoprenol, 8 ng/mg (dw; 2 µg/mL Dox) to 339 ng/mg (dw; 1 µg/mL Dox) of farnesol, and 2 ng/mg (dw; 2 µg/mL Dox) to 8 ng/mg (dw; 1 µg/mL Dox) of geranylgeraniol. The use of alkaline phosphatase (compare Figure 2III) capturing pyrophosphorylated intermediates did not influence terpene levels.

**Figure 9:**
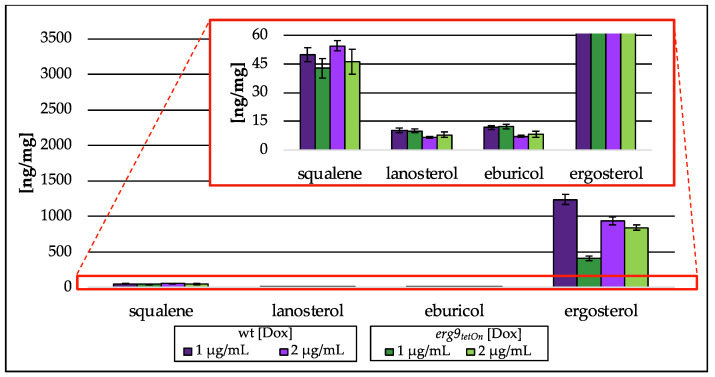
Amount of squalene and intermediates of the post-squalene pathway in ng per mg biomass (dry weight) extracted from the conditional *erg9_tetOn_* strain cultured with different concentrations of doxycycline (Dox; 1 µg/mL, 2 µg/mL). In the red box, the zoomed *y*-axis is depicted. Error bars represent the standard deviation from three biological replicates, each analyzed as technical duplicates.

Lanosterol and eburicol, representative sterols of the post-squalene pathway, were hardly affected, whereas ergosterol levels were decreased to 410 ng/mg (dw; 1 µg/mL Dox)-839 ng/mg (dw; 2 µg/mL Dox; Figure 9), which is a decrease of 67% (1 µg/mL Dox)-10% (2 µg/mL Dox) compared to their respective wt strain. In addition, the amount of squalene was reduced to 46 ng/mg (dw; 1 µg/mL Dox)/43 ng/mg (dw; 2 µg/mL Dox), a decrease of 14% (1 µg/mL Dox)/15% (2 µg/mL Dox) in relation to the wt. The associated *A. fumigatus* wt strain did not contain any isoprenoids of the isoprenoid pathway of ergosterol biosynthesis above the lower limit of quantification. 

#### 3.3.2. Analysis of the Conditional *erg1* Strain

**Figure 10:**
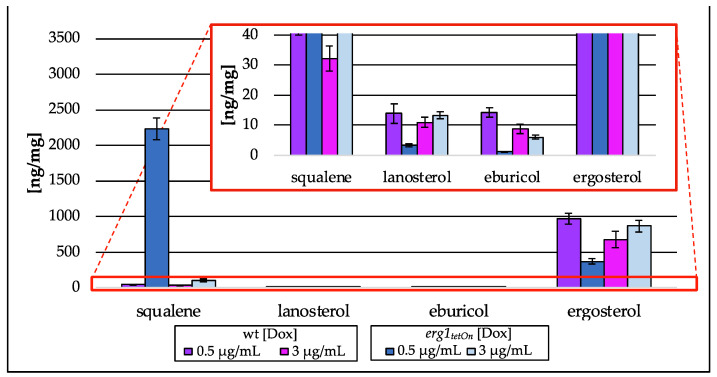
Amount of squalene and intermediates of the post-squalene pathway in ng per mg biomass (dry weight) extracted from the conditional *erg1_tetOn_* strain cultured with different concentrations of doxycycline (Dox; 0.5 µg/mL, 3 µg/mL). In the red box, the zoomed *y*-axis is depicted. Error bars represent the standard deviation from three biological replicates, each analyzed as technical duplicates.

The growth analysis of the conditional *erg1* mutant strain also revealed a growth defect under fully repressed conditions (Supplementary Material Figure S1). Low doxycycline concentrations enabled this mutant to grow, even though growth was reduced when compared to the wt or the mutant under fully induced conditions. The reduced growth is accompanied by a Dox-dependent accumulation of squalene and a decrease in eburicol (Figure 10). The squalene levels increased 3–50-fold (100 ng/mg (dw; 3 µg/mL Dox)-2236 ng/mg (dw; 0.5 µg/mL Dox)), compared to the wt strain 32 ng/mg (dw; 3 µg/mL Dox)-45 ng/mg (dw; 0.5 µg/mL Dox), whereas eburicol levels were between 6 ng/mg (dw; 3 µg/mL Dox) and 1 ng/mg (dw; 0.5 µg/mL Dox) compared to 9 ng/mL (dw; 3 µg/mL Dox) and 14 ng/mg (dw; 0.5 ng/mL Dox) in wt, a decrease of 32% (3 µg/mL Dox)–92% (0.5 µg/mL Dox). Lanosterol and ergosterol amounts, however, slightly increased when the mutant strain was exposed to high concentrations of Dox (3 µg/mL), whereas a low Dox concentration (0.5 µg/mL) resulted in a 76% decrease in lanosterol amount (3 ng/mg; dw) and 62% decrease in ergosterol amount (370 ng/mg; dw). None of the isoprenoid pathway intermediates were detected in samples from the *erg1* mutant strain as well as the associated wt strain.

#### 3.3.3. Analysis of *erg9*/*erg1* Culture Supernatants

**Figure 11:**
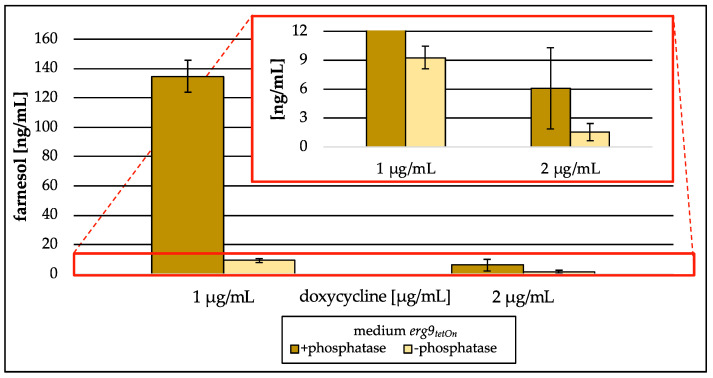
Amount of farnesol in ng per mL medium (freeze-dried) gained from the conditional *erg9_tetOn_* strain cultured with different concentrations of doxycycline (1 µg/mL, 2 µg/mL). In the red box, the zoomed *y*-axis is depicted. Error bars represent the standard deviation from three biological replicates.

Analysis of lyophilized culture supernatants did not show any intermediates of the isoprenoid pathway in medium collected from the conditional *erg1* mutant strain when the corresponding gene was downregulated. The culture supernatant gained from the conditional *erg9* mutant strain during low induction (1 µg/mL/2 µg/mL Dox), however, showed high levels of farnesol (Figure 11). This farnesol was generated from farnesyl pyrophosphate by enzymatic deconjugation during sample preparation (compare Figure 2III). If the enzymatic deconjugation was conducted as a part of sample preparation, 135 ng/mL of medium farnesol was detected in the culture supernatant of the *erg9* mutant strain incubated with 1 µg/mL of Dox, whereas without the enzymatic step, only 9 ng/mL of medium were detected. Also, the culture supernatant harvested from the *erg9* mutant strain incubated with 2 µg/mL of Dox contained farnesyl pyrophosphate, which was analyzed as farnesol. The amount was 2 ng/mL of medium without the use of alkaline phosphatase and 6 ng/mL of medium when the enzyme was used. Further isoprenoids could not be detected. 

#### 3.3.4. Terbinafine Treatment of the Wild Type Strain

**Figure 12:**
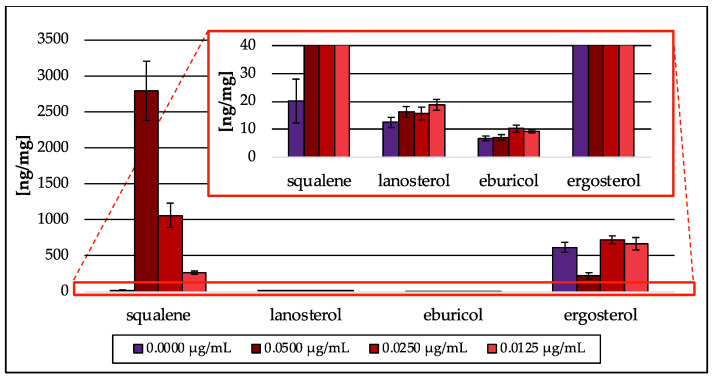
Amount of squalene and intermediates of the post-squalene pathway in ng per mg biomass (dry weight) extracted from *A. fumigatus* wt strain treated with different concentrations of terbinafine. In the red box, the zoomed *y*-axis is depicted. Error bars represent the standard deviation from three biological replicates, each analyzed as technical duplicates.

As proof of concept, the Erg1 inhibitor terbinafine was tested [11,29]. Terbinafine treatment resulted in a concentration-dependent accumulation of squalene (270–2793 ng/mg; dw) and a severe decrease in ergosterol (228 ng/mg; dw) at the highest (0.0500 µg/mL) terbinafine concentration tested (Figure 12). Exposure of wt to the two lower terbinafine concentrations (0.0250 and 0.0125 µg/mL) neither led to a decrease in ergosterol nor the reduction in its precursor sterol lanosterol and eburicol. Intermediates from the isoprenoid pathway could not be detected. Taken together, terbinafine treatment resulted in a similar sterol pattern as it was observed in the *erg1* repression model (see Figure 10).

## 4. Discussion

A novel gas chromatography–mass spectrometry (GC-MS) method for the analysis of intermediates of the isoprenoid pathway was developed. The method was optimized for the extraction of pyrophosphorylated terpenes from different fungal sources. Covering a calibration range from 2.5/25 to 1000 ng/mL (terpenes/squalene), the approach is sensitive to measuring small changes in levels of isoprenoid biosynthesis intermediates. In addition, signals are measured with a high accuracy (low, medium and high level: 93–112%; LLOQ: 82–115%) and selectivity (low, medium and high level: <6%; LLOQ: <14%). To our knowledge, this is the first GC-based approach covering the whole isoprenoid pathway including the isomers dimethylallyl pyrophosphate and isopentenyl pyrophosphate in one straightforward protocol. By combining this new isoprenoid assay (five involved enzymes including Bts1) with the established post-squalene ergosterol biosynthesis assay (Müller et.al. [9,28]; ten involved enzymes), effects on 15 different enzymes can be covered from one sample. This will make it easier to identify mechanisms of action for new potential antifungals affecting ergosterol biosynthesis. Further advantages are the small amount of sample matrix that is required (dw; 5 ± 0.2 mg), as well as the short time of analysis (23 min cycle time). The only disadvantage of the approach is its limitation to the intermediates of the isoprenoid pathway. A potential inhibition of mevalonate pathway enzymes can only be assumed as a change in the downstream sterol pattern but cannot be assigned to a specific enzyme. Up until now, only liquid chromatography (LC)-based approaches have been available for the analysis of mevalonate pathway intermediates (Table 1). In LC methods, mono- and di-phosphates can be distinguished, whereas the simultaneous analysis of the isomers isopentenyl pyrophosphate and dimethylallyl pyrophosphate is not possible [39].

The analysis of the *A. fumigatus* mutant strain revealed that the main part of the detected terpenes was already intracellularly available as free alcohols and enzymatic deconjugation could not increase the detected terpene levels (Section 3.3.1). Our approach confirmed that only negligible amounts of farnesyl pyrophosphate were available in cells from the *erg9* mutant, whereas the main part was already dephosphorylated (Section 3.3.1). Besides farnesol, small amounts of geranylgeraniol and isoprenol were also found. Those analytes could be detected because of a strong farnesyl pyrophosphate accumulation, which increased the production of geranylgeranyl pyrophosphate and induced an accumulation of the upstream substrate isopentenyl pyrophosphate (Figure 1). Even though the pyrophosphates were suspected to have accumulated, it must be assumed that intracellular dephosphorylation occurred.

The analysis of the post-squalene pathway showed a slight reduction in squalene levels as well as a Dox-dependent decrease in ergosterol in the mutant strain, which is the expected consequence of an *erg9* downregulation. The generated sterol pattern therefore is in accordance with the results from the growth test of the conditional *erg9* strain (Supplementary Material Figure S1). 

In the *erg1* mutant, no isoprenoid pathway intermediate could be detected above the limit of detection, even though upstream intermediates were expected as a secondary effect of squalene accumulation in the same way geranylgeraniol and isoprenol were found in the *erg9* mutant. Post-squalene pathway analysis showed increased squalene levels and decreased ergosterol amounts, which could be expected due to *erg1* downregulation. This outcome is similar to that observed for terbinafine treatment of the (*A. fumigatus*) wt strain (Section 3.3.4). Squalene levels in terbinafine-treated cells were increased and are in line with reduced amounts of ergosterol.

Surprisingly, the analysis of lyophilized growth medium revealed high amounts of farnesyl pyrophosphate, which were quantified as farnesol–*t*BDPS ether (Section 3.3.3). In comparison to an approach where the enzymatic deconjugation step was skipped, farnesol levels in culture supernatant were 4–14-fold higher when alkaline phosphatase was used. This indicates that farnesyl pyrophosphate is the preferred form to be extruded when increased intracellular levels occur. In contrast, farnesol is the preferred substrate accumulating intracellularly. Those results are contrary to the findings of Song [36], who identified increased farnesyl pyrophosphate levels in cells from *S. cerevisiae erg9* mutant strains and assumed higher farnesol levels extracellularly in the culture medium [18]. 

The intra- and extracellular appearance of farnesol is of great interest because of its diverse attributes. In its role as a quorum-sensing molecule, farnesol is used to signal extracellularly with the environment, including other fungi and bacteria, and therefore has effects on virulence and biofilm formation [55,56]. Several reports [55,57] suggest that due to its immunomodulatory properties, the molecule promotes disease and even increases mortality during systemic *C. albicans* infection. On the other hand, synergistic effects with several antifungal drugs were identified, which are caused by a farnesol-mediated inhibition of the ABC drug transporters [58].

Besides the vague role of farnesol in fungal cells that must be further investigated, the interest in the whole isoprenoid pathway should be increased. Based on the essential role of isoprenoid pathway genes, the associated enzymes are promising targets for drug development. Since the isoprenoid pathway is part of both human and fungal metabolism, one of the largest challenges will therefore be the selectivity of potential drug candidates in fungal cells, as well as the investigation of the effect of isoprenoids on the host biosystem. However, the success of the imidazoles/triazoles, which also target both the mammalian and fungal sterol C14-demethylase, is indisputable. Expanding the established test system on mammalian cells should be a promising option to confirm selectivity.

## Figures and Tables

**Table 1:** Overview of methods described in the literature for the analysis of intermediates of the pre-squalene pathway: dimethylallyl pyrophosphate (DMAPP), farnesyl pyrophosphate (FPP), geranyl pyrophosphate (GPP), geranylgeranyl pyrophosphate (GGPP), isopentenyl pyrophosphate (IPP), mevalonate phosphate (MVP), mevalonate pyrophosphate (MVPP), mevalonic acid (MVA), squalene (SQ); n.d. not determined.

Reference	Analytical System	Analytes	Biological Matrix	Quantification Limit (ng/mL)
Bruenger, E. et al., 1988 [35]	TLC-scintillation	IPP, FPP	mice liver	n.d.
McTaggart, F. et al., 1996 [34]	HPLC-scintillation	IPP, GPP, FPP, GGPP, SQ	rat hepatic microsomes	n.d.
Song, L. 2003 [36]	GC-MS	FPP	cultured fungal cells	n.d.
Tong, H. et al., 2005 [37]	HPLC-fluorescence	FPP, GGPP	cultured human cell lines	n.d.
Hooff, G. et al., 2008 [38]	HPLC–MS/MS	FPP, GGPP	human brain cells	10 (FPP) 50 (GGPP)
Vallon, T. et al., 2008 [22]	GC-MS	FPP, GGPP	bacteria	77 (FPP) 90 (GGPP)
Henneman, L. et al., 2008 [39]	HPLC–MS/MS	MVA, MVP, MVPP, IPP/DMAPP, GPP, FPP, GGPP	cultured human cell lines	618 (MVA) 951 (MVP) 1285 (MVPP) 31 (IPP/DMAPP) 103 (GPP) 42 (FPP) 59 (GGPP)
Huang, B. et al., 2011 [33]	GC-MS	GPP, FPP, GGPP, SQ	cultured fungal cells	218 (GPP) 143 (FPP) 21 (GGPP) 6 (SQ)
Rodriguez, S. et al., 2014 [17]	GC-MS	MVA, FPP, SQ	cultured fungal cells	500 (MVA) 500 (FPP) 500 (SQ)
Chhonker, Y. et al., 2018 [40]	HPLC–MS/MS	GPP, FPP, GGPP	cultured human cell lines and human plasma	0.04 (GPP) 0.04 (FPP) 0.04 (GGPP)
Castaño-Cerezo, S. et al., 2019 [41]	HPLC–HRMS	MVA, MVP, MVPP, IPP/DMAPP, GPP, FPP, GGPP	cultured fungal cells	22 (MVA) 2 (MVP) 3 (MVPP) 5 (IPP) 3 (DMAPP) 16 (GPP) 8 (FPP) 36 (GGPP)

**Table 2:** Analytical details of the analyzed isoprenoid *t*BDPS ethers and squalene. Absolute retention time (RT); relative retention time (RRT); in bold, quantifier ions.

Trivial Name	RT (min)	RRT	Qualifier and Quantifier Ions (*m*/*z*)
isoprenol	9.90	0.707	69, **225**, 267
prenol	9.96	0.712	69, 189, **267**
geraniol	11.35	0.811	69, **335**, 392
squalene	12.03	0.860	**69**, 81, 410
farnesol	12.73	0.910	**69**, 203, 403
1-heptadecanol (IS_Iso_)	14.00	1.000	71, 123, **437**
geranylgeraniol	15.26	1.090	**69**, 81, 471

**Table 3:** Analytical details of the analyzed sterol TMS ethers according to Müller et al. [9,28]. Absolute retention time (RT); relative retention time (RRT); in bold, quantifier ions.

Trivial Name	RT (min)	RRT	Qualifier and Quantifier Ions (*m*/*z*)
cholestane (IS_Sterol_)	11.75	1.000	203, **217**, 357
lanosterol	16.83	1.440	241, **393**, 498
eburicol	17.46	1.501	**407**, 498, 512
ergosterol	15.54	1.329	337, **363**, 378

**Table 4:** Recovery and SD values of spiked isoprenoids extracted from *S. cerevisiae*. Values were normalized using matrix-matched standards (1000 ng/mL). SD is the standard deviation from six technical replicates.

	Isoprenol	Prenol	Geraniol	Squalene	Farnesol	Geranyl- Geraniol
Recovery (%)	88	83	74	73	67	42
SD (%)	5	4	3	5	8	7

**Table 5:** Overview of validation data; n.d., not determined.

Validation Parameter		Isoprenol	Prenol	Geraniol	Squalene	Farnesol	Geranyl- Geraniol
Linearity (ng/mL)	Calibration range	2.5–1000	2.5–1000	2.5–1000	25–1000	2.5–1000	2.5–1000
Average Accuracy (%)	Day 1	91	90	104	98	109	111
	Day 2	92	87	94	83	97	105
	Day 3	100	104	104	100	107	109
Precision (%)	Day 1	3	4	3	5	4	3
	Day 2	5	4	6	6	3	3
	Day 3	4	3	3	4	3	3
Matrix factor	Low QC	1.11	1.03	1.05	1.66	1.99	1.21
	High QC	1.01	1.02	0.99	1.16	1.03	1.05
Carry over (%)		35	33	29	90	61	49
Dilution Integrity		confirmed	confirmed	confirmed	confirmed	confirmed	confirmed
Stability		stability was tested after 5/30 days—storage should be performed in a freezer (−20 °C)					
Recovery (%)	*S. cerevisiae*	n.d.	107	n.d.	n.d.	133	44
	*A. fumigatus*	n.d.	85	n.d.	n.d.	109	37

## Data Availability

Data available from the corresponding author.

## Supplementary Materials

The following supporting information can be downloaded at: https://www.mdpi.com/article/10.3390/jof9070768/s1, Figure S1: Doxycycline-dependent growth of the conditional *erg9* and *erg1* strains. Validation results included. Figure S2: Selected ion chromatogram of quantifier ions at different concentrations. Figure S3: Single-ion-monitoring spectra of the isoprenoid *t*BDPS ethers, the internal standard *t*BDPS ether and squalene. Figure S4: Full-scan spectra of the isoprenoid *t*BDPS ethers, the internal standard *t*BDPS ether and squalene. Figure S5: Investigation of matrix effects comparing signal areas of low- and high-QC samples in *n*-hexane and sample matrix. Figure S6: Sample stability over 30 days comparing stability on days 0, 5 and 30. Figure S7: Recovery of three representative pyrophosphates from *S. cerevisiae* and *A. fumigauts* matrix. Table S1: Evaluation of method accuracy measured on three subsequent days. Table S2: Evaluation of method precision measured on three subsequent days. Table S3: Matrix effects determined at two different concentrations and the associated standard deviation. Table S4: Carry over. Table S5: Standard deviation as additional criterion for recovery.
