# Supplementary material for: Quantifying Isoprenoids in the Ergosterol Biosynthesis by Gas Chromatography–Mass Spectrometry
Source: J Fungi (Basel). 2023 Jul 20;9(7):768. doi: 10.3390/jof9070768 (PMC10381423; doi:10.3390/jof9070768)
Supplement: Supplementary file 1 [file jof-09-00768-s001.zip › jof-2451533-supplementary.pdf]

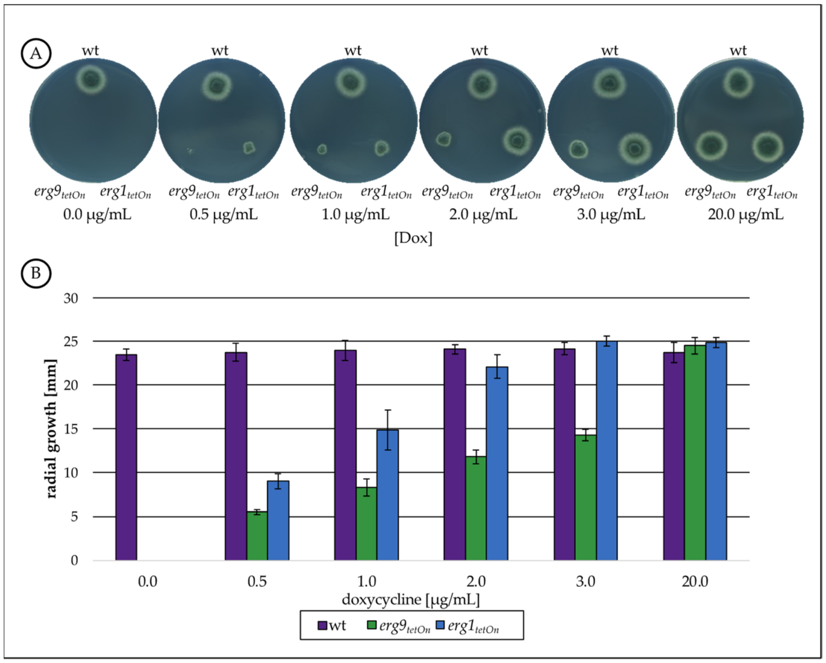

Figure S1. Doxycycline-dependent growth of the conditional *erg9* and *erg1* strains. 1,500 conidia of the indicated strains were spotted on AMM agar plates with the indicated concentration of doxycycline [Dox]. Plates were then incubated at 37 °C. A: Representative photos were taken after 48 h of incubation. B: The diameters of three colonies per strain and condition was measured after 48 h and plotted in the graph. The error bars indicate standard deviations.

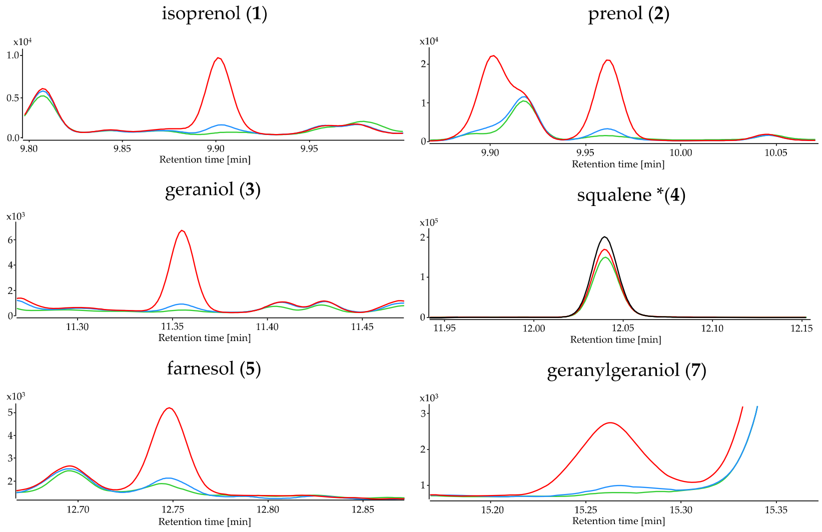

Figure S2. Selected ion chromatogram of quantifier ions at different concentrations analyzed from matrix matched samples (*S. cerevisiae*). Green: matrix blank; blue: lower limit of quantification (LLOQ) 2.5 ng/mL; red: 25 ng/mL; black: 100 ng/mL; * Squalene is an endogenous analyte; no squalene free matrix was available.

isoprenol (**1**)

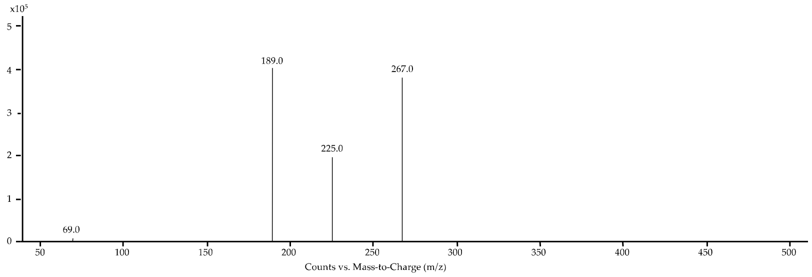

prenol (**2**)

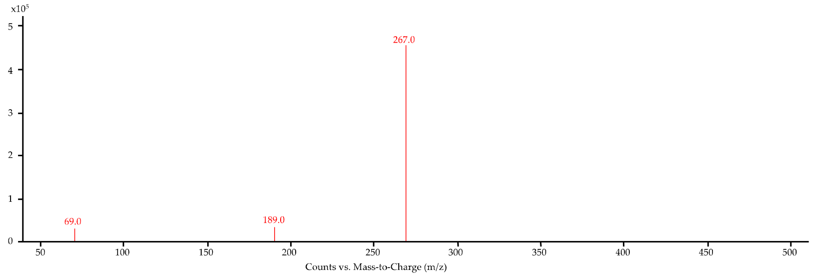

geraniol (**3**)

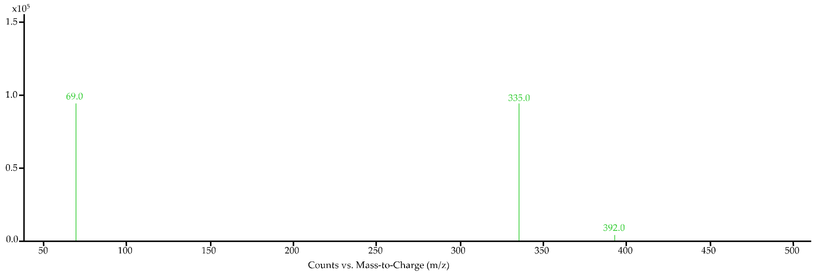

squalene (**4**)

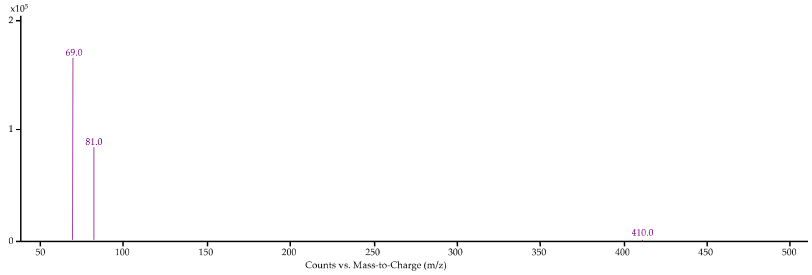

farnesol (**5**)

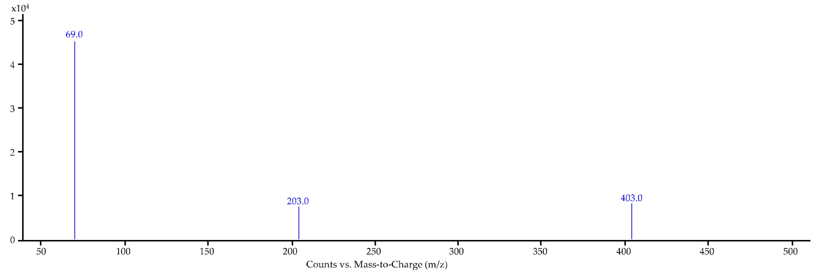

Figure S3. (continued)

IS (**6**)

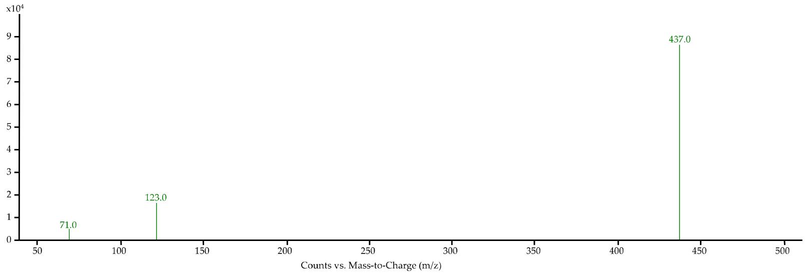

geranylgeraniol (**7**)

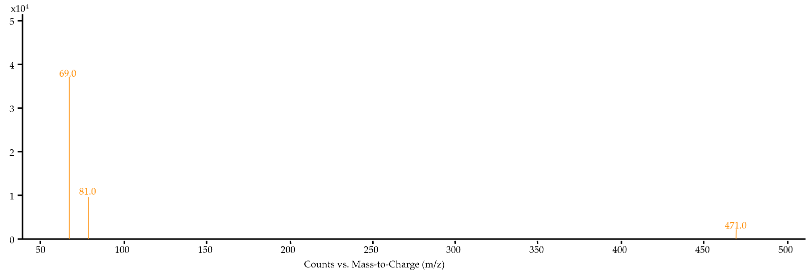

Figure S3. Single ion monitoring (SIM) spectra of the isoprenoid *t*BDPS ethers, the internal standard (1-heptadecanol; IS) *t*BDPS ether and squalene.

isoprenol (**1**)

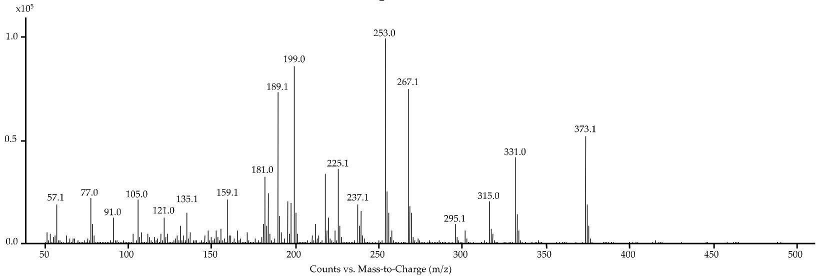

prenol (**2**)

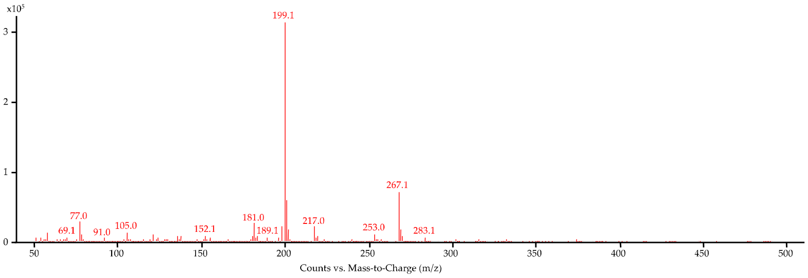

geraniol (3)

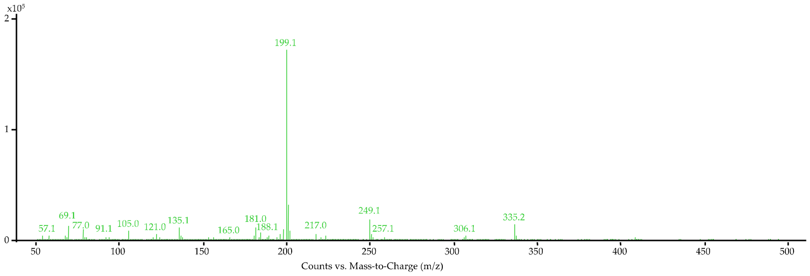

Figure S4. (continued)

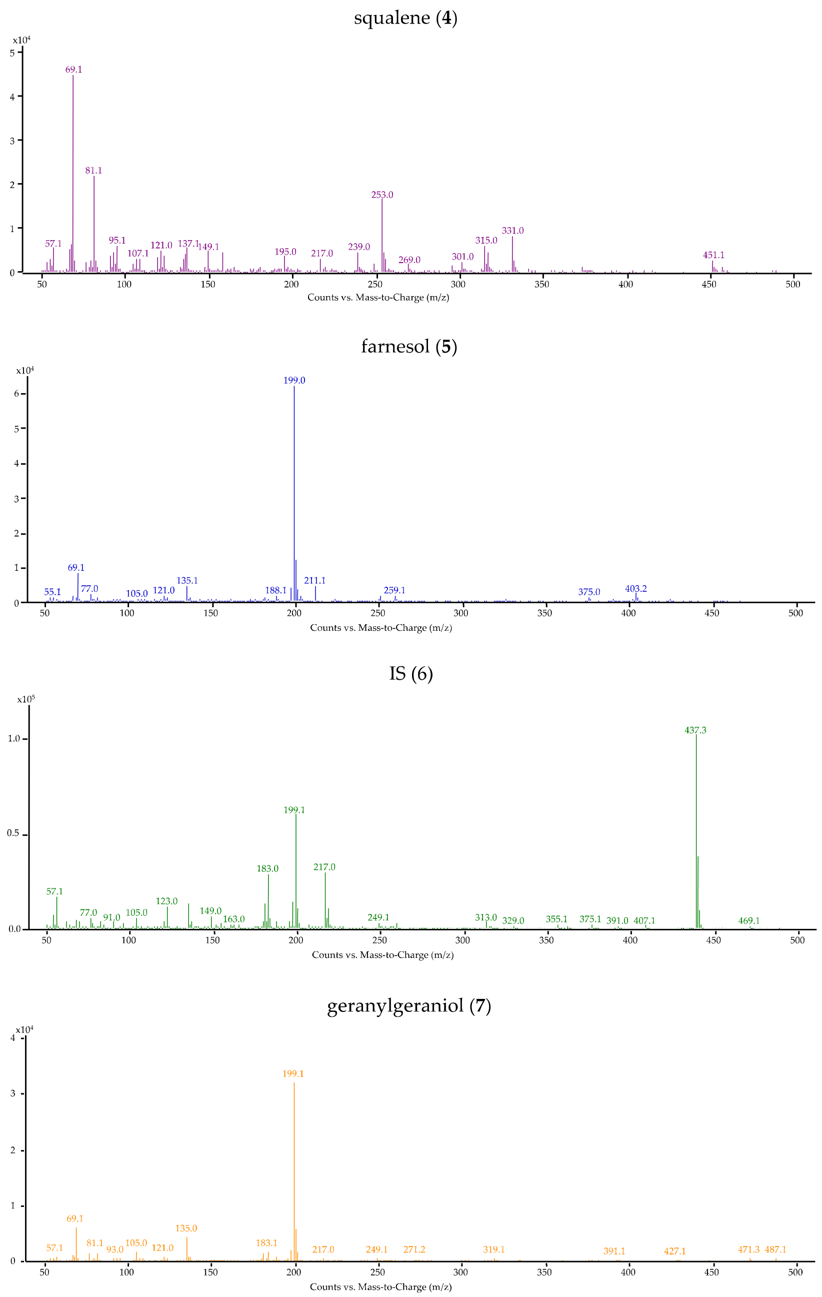

Figure S4. Full Scan spectra *m/z* 50-500 of the isoprenoid *t*BDPS ethers, the internal standard (1-heptadecanol; IS) *t*BDPS ether and squalene.

S1. Method validation

Method validation was performed based on the European Medicines Agency (EMEA) guideline on bioanalytical method validation EMEA/CHMP/EWP/192217/2009 [1].

1.1 Selectivity

Selectivity was confirmed by the relative retention time (RRT) of each analyte relating to the internal standard 1-heptadecanol (IS_{Iso}) and using three characteristic ions, two qualifiers and one quantifier ion (*Article*: **Table 2**, **Table 3**).

S1.2 Linearity

Linearity of the calibration curves were determined by measuring spiked samples (matrix matched). Nine levels (2.5, 5.0, 10, 25, 50, 100, 250, 500 and 1,000 ng/mL) with a consistent concentration of IS_{Iso} (5 µg/mL) were analyzed in triplicates. Peak area ratios from quantifier ions of analytes and IS_{Iso} were plotted against the corresponding analyte concentration. The calibration curve was weighted (1/x) and used to back-calculate the individual concentration. The back-calculated concentration was further used to determine the standard deviation (SD), which should be within ±15% (lower limit of quantification (LLOQ) ±20%) of the nominal concentration. At least 75% of the calibration standards and 50% of the used replicates must fit those parameters.

S1.3 Lower limit of quantification (LLOQ)

The lower limit of quantification (LLOQ) is defined as the lowest level of the calibration curve that can be quantified reliably. As additional criteria the parameters for accuracy and precision (*Supplementary material*: Chapter 2.2) were considered, whereas contrary to the guideline a peak area five-times higher than a blank sample (matrix blank) was not taken into account due to the endogeneity of squalene and interferences from consumables (*Supplementary material*: Chapter 2.1).

S1.4 Accuracy and method precision

Accuracy and method precision were determined at LLOQs, low level (3×LLOQ), medium level (500 ng/mL) and high level (1,000 ng/mL) level. Therefore, sample matrix was prepared as described in *Article:* Chapter 2.5.3 and pooled. The quality control (QC) samples were prepared by spiking matrix with 10 µL of an appropriate stock solution containing a mixture of all analytes before derivatization (n=6). To approve accuracy, the analyte signals were used to back-calculate the concentration of each level based on the calibration curve which was prepared independently from the QC samples with separately prepared stock solutions. The calculated concentration was divided by the nominal concentration. The SD of every level was calculated from the average relative response. Between-run accuracy and between-run precision were determined by repeating the analysis on three consecutive days.

S1.5 Instrument precision

The instrument precision was determined by calculating the SD for all analytes at different concentrations (LLOQ, low QC, medium QC, and high QC) after sixfold injection.

S1.6 Investigation of matrix effects

Matrix effects were determined by comparing spiked samples prepared in matrix to samples prepared in *n*-hexane at two different concentrations (low and high; n=6). Matrix was prepared as described in *Article:* Chapter 2.5.3. For the evaluation of matrix effects the EMEA guideline recommends the use of blank matrix but due to high variations in endogenous squalene concentrations, pooled matrix was used [1]. The overall SD for the calculated concentration should not be greater than 15%.

S1.7 Carry–over

Carry–over was determined by measuring matrix blanks (n=3), subsequently after analyzing standards at 1,000 ng/mL. The resulting signal areas were compared to the average areas of matrix blanks (n=6).

S1.8 Dilution integrity

Dilution integrity was confirmed by preparing a stock solution containing 2,000 ng/mL of all analytes in matrix. This concentration was twofold higher than the upper limit of quantification which is the highest level of the calibration curve (1,000 ng/mL). From this stock solution 500, 250 or 25 µL were diluted with freshly prepared sample extract (*Article:* Chapter 2.5.3) as a solvent (n=6) to generate dilution integrity samples of 1,000 µL (corresponding to a dilution factor of 2, 4 and 40). Using a calibration curve, the actual dilution integrity sample concentration was back–calculated and compared to the nominal concentration.

S1.9 Stability

Long time stability was determined using six matrix matched samples of low (3×LLOQ) and high level that were stored under two different conditions. One batch of samples was stored at room temperature (RT) whereas the other batch was stored at -20 °C. Samples were analyzed on days 0, 5 and 30. The relative change of analyte concentration was determined by comparing the relative responses (area analyte/area IS_{Iso}) of each analyte with the values from day 0.

S1.10 Recovery

Recovery was determined on equimolar concentrations to low, medium, and high QC samples, due to the use of pyrophosphates instead of the free alcohols. The analyses were carried out on three different representative analytes: dimethylallyl pyrophosphate (C_5), farnesyl pyrophosphate (C_{15}), and geranylgeranyl pyrophosphate (C_{20}). The prepared samples were analyzed (n=6) and compared to spiked QC samples of the same concentration.

S2. Validation results

S2.1 Selectivity

No interfering peaks from endogenous compounds were detected in the range of the retention times of the analyzed isoprenoid *t*BDPS ethers and squalene, until the samples were spiked. Only squalene as an endogenous matrix component was present in all samples that contain matrix. For the derivatized isoprenol, prenol and farnesol *t*BDPS ethers interferences from consumables were detected (**Figure S2**). Those interferences could be avoided by switching from 2 mL plastic microcentrifuge safe–lock tubes to glass vials. Because plastic microcentrifuge safe–lock tubes were essential for further sample preparation, they cannot be exchanged and interferences from these consumables must be considered. A matrix matched standard preparation considering these interferences, is therefore mandatory.

S2.1 Linearity and LLOQ

For all analytes a linear fit could be determined ($R^2 \geq 0.994$). The linear range for isoprenol, prenol, geraniol, farnesol and geranylgeraniol was 2.5 to 1,000 ng/mL. The linear range for squalene was 25 to 1,000 ng/mL because endogenous squalene was detected in blank matrix. According to the EMEA guideline the lowest level of the calibration range which can be quantified with an acceptable accuracy (±20%) and precision (±20%) is defined as the lower limit of quantification (LLOQ) [1].

S2.2 Accuracy, method precision and injection precision

For evaluation of accuracy six QC samples of four concentrations LLOQ (2.5/25 ng/mL), low (7.5/75 ng/mL), medium (500 ng/mL), and high (1,000 ng/mL) were analyzed (**Table S1**). The analyte peak area of each sample was used to calculate the sample concentration and was further compared to the nominal concentration (**Table S1**, **Day 1**, **Day 2**, **Day 3**), whereas the three days average was the mean value from three independent accuracy measurements (**Table S1, Average**). The average concentration should be within 85% to 115% at the low, medium, and high QC, whereas for the LLOQ 80% to 120% were accepted. All analyte levels fulfilled the criteria for accuracy (**Table S1**, **Day 1**, **Day 2**, **Day 3**). Only geranylgeraniol reached the limits slightly with accuracy values of 120% at the LLOQ on day 1 and 119% at the high concentration on day 3 (**Table S1 Day 1, Day 3**). To confirm between–day accuracy the test for accuracy was performed on three consecutive days. The average accuracy of three different batches was in between 93% (prenol) and 112% (geranylgeraniol) at concentrations above the LLOQ whereas at the LLOQ variations were between 82% (squalene), and 115% (geranylgeraniol) were detected (**Table S1, Average**).

Method precision was evaluated within the same batch of samples (**Table S1**). Method precision was calculated as the SD of the individual analyte responses on different days. The precision at the LLOQ did not exceed a SD of 14% (squalene) which was below the limit of 20% (**Table S2**, **Day 2**). Also, in low (6%; **Table S2**, **Day 3**; geraniol), medium (6%; **Table S2**, **Day 2**; geraniol), and high (4%; **Table S2**, **Day 3**; farnesol and geranylgeraniol) QCs met the criterion of SD < 15%.

Injection precision was determined at four levels (LLOQ, low, medium, high). The highest SDs were calculated for the analytes

isoprenol, prenol, and geranylgeraniol at LLOQ with values between 6 and 10% (sixfold injection). At low QC the maximum SD of 5% was calculated for geranylgeraniol, whereas in the medium and high QCs none of the analytes exceeded a SD > 4% (data not shown).

Table S1. Evaluation of method accuracy measured on three subsequent days (n=6) including the average accurracy.

Accuracy [%]						
	isoprenol	prenol	geraniol	squalene	farnesol	geranylgeraniol
**Day 1**						
LLOQ	87	81	114	99	115	120
low	90	88	105	112	104	106
medium	95	97	99	93	111	107
high	93	95	97	88	107	109
**Day 2**						
LLOQ	94	80	100	63	91	116
low	94	87	89	89	90	96
medium	89	90	92	90	102	101
high	89	91	94	90	104	108
**Day 3**						
LLOQ	90	100	99	85	103	109
low	102	103	104	104	105	99
medium	102	104	102	102	108	109
high	107	109	109	107	113	119
**Average**						
LLOQ	90	87	104	82	103	115
low	95	93	100	102	100	100
medium	95	97	98	95	107	106
high	97	98	100	95	108	112

Table S2. Evaluation of method precision measured on three subsequent days (n=6).

Precision [%]						
	isoprenol	prenol	geraniol	squalene	farnesol	geranylgeraniol
**Day 1**						
LLOQ	2	4	3	11	5	3
low QC	3	4	3	4	5	2
medium QC	4	4	3	3	2	3
high QC	3	3	4	4	3	3
**Day 2**						
LLOQ	8	4	10	14	2	3
low QC	4	2	4	5	4	5
medium QC	5	5	6	2	3	3
high QC	3	3	3	3	2	3

Table S2. (continued)

Precision [%]						
	isoprenol	prenol	geraniol	squalene	farnesol	geranylgeraniol
			Day 3			
LLOQ	6	2	2	9	4	3
low QC	4	3	6	3	2	5
medium QC	3	3	2	1	2	2
high QC	3	3	3	3	4	4

S2.3 Matrix effects

The low QC and high QC samples were prepared in pooled sample matrix to generate a stable level of squalene because no squalene free matrix was available. The matrix effects were determined by comparing the peak areas of spiked samples in matrix to samples prepared in *n*-hexane (**Table S3, Matrix factor**). For the analytes farnesol, squalene, and geranylgeraniol concentration dependent matrix effects which were stronger in low QC samples, were determined. Peak areas were decreased in *n*-hexane (**Figure S5)**. In contrast, the internal standard peak areas were increased when *n*-hexane was used. According to the guideline the SD of the peak area should be <15%, which is fulfilled for all analytes at both concentrations (**Table 3 RSD**). Only squalene (15%) was at the upper limit of the criterion at the low QC. Due to the detected matrix effects, calibration standards should be prepared in sample matrix, as already mentioned in *Supplementary material*: Chapter 2.1

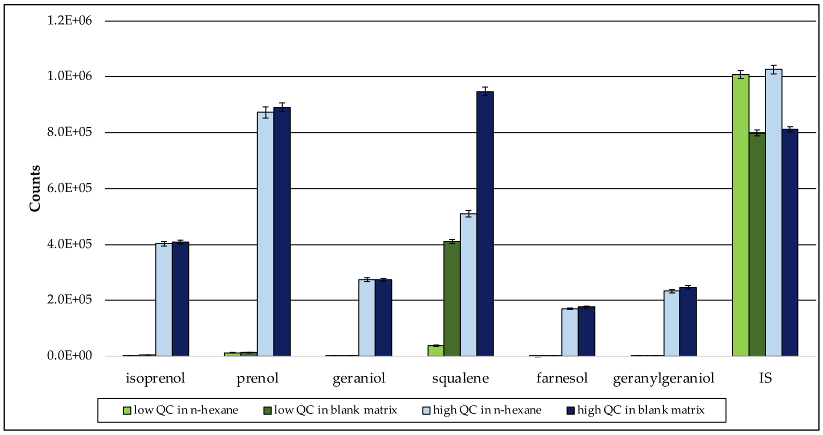

Figure S5. Peak areas of low and high QC samples in *n*-hexane and sample matrix. Error bars represent the standard deviation (n=6).

Table S3. Matrix effects determined at two different concentrations and the associated RSD (n=6).

Matrix factor							
	isoprenol	prenol	geraniol	squalene	farnesol	geranylgeraniol	IS
low QC	1.11	1.03	1.05	1.66	1.99	1.21	0.79
high QC	1.01	1.02	0.99	1.16	1.03	1.05	0.79
				RSD [%]			
low QC	5	3	5	15	8	9	1

Table S3. (continued)

	RSD [%]						
	isoprenol	prenol	geraniol	squalene	farnesol	geranylgeraniol	IS
high QC	2	2	2	2	2	2	1

S2.4 Carry-over

By analyzing blank matrix samples after high concentration samples, the influence of carry-over (CO) was investigated. According to the guideline, signals from blank samples injected right after high concentration samples (high QC; 1,000 ng/mL) should not exceed 20% of the signal area of LLOQ samples (5% for the IS_{Iso}). Due to the interferences induced by consumables for the signals of prenol, isoprenol and farnesol (*Supplementary material:* Chapter 2.1) as well as the matrix composition including high amounts of squalene, the parameter could not be fulfilled according to the guideline, even though there was no visible CO. For geraniol (29%) and geranylgeraniol (49%) that were not affected by any interfering signals CO could be seen, whereas the internal standard (IS_{Iso}) was not affected (2%) (**Table 4**). To test this parameter on the remaining analytes (isoprenol, prenol, squalene and farnesol), the approach was adapted (see *Supplementary material*: Chapter 1.7). The variation between matrix blanks measured after high QC samples and independent matrix blanks (matrix blanks measured prior to the first samples that can therefore not be affected by CO) was determined. The biggest differences were detected for isoprenol (13%), prenol (19%), and the IS_{Iso} (15%), whereas for geraniol, squalene, farnesol, and geranylgeraniol values below 10% were calculated (**Table S4; Carry over (adapted)**). This means that according to the adapted approach, the difference was below 20% and therefore samples analyzed subsequently after high concentration standards are not significantly affected by a previous high concentration sample.

Table S4. Carry over determined according the EMEA guideline, as well as an adapted approach (n=3).

	Carry-over (EMEA) [%]						
	isoprenol	prenol	geraniol	squalene	farnesol	geranylgeraniol	IS
	35	33	29	90	61	49	2
	**Carry-over (adapted) [%]**						
relative change	13	19	9	2	7	4	15

S2.5 Dilution integrity

Usually, calibration ranges are selected to cover the expected concentration range. Nevertheless, biological samples can also contain analyte levels above the expected amounts. For this reason, dilution should be possible in a linear range to fit the sample concentration levels to the calibration range. The parameter of dilution integrity was determined diluting a stock solution of 2,000 ng/mL (all analytes), which was above the highest calibration level (1,000 ng/mL), to generate samples with 1,000 ng/mL (factor 2) 500 ng/mL (factor 4) and 50 ng/mL (factor 40). None of the prepared concentration levels (n=6) varied more

than 15% from the nominal concentration and therefore showed a linear correlation (data not shown).

S2.6 Stability

The evaluation of sample stability was performed analyzing samples of low (3×LLOQ) and high (1,000 ng/mL) concentrations (n=6) repeatedly after storing under different storing conditions (RT, -20 °C; **Figure S6**). After five days mostly all samples showed SD values ±15%. Only replicates of squalene (3×LLOQ) stored at room temperature (day 5) exceeded the parameters with a relative response of 127% compared to their reference value (day 0). After a storage period of 30 days the samples stored at room temperature could not be quantified reliably anymore. For samples at 3×LLOQ only two out of six analytes showed the expected response (low QC (RT) day 30), whereas high concentrations could be analyzed more reliably. Samples stored in a freezer were more stable and showed lower SD values. Only two (isoprenol and farnesol) out of six low QC analytes did not meet the stability criteria. Therefore, the samples

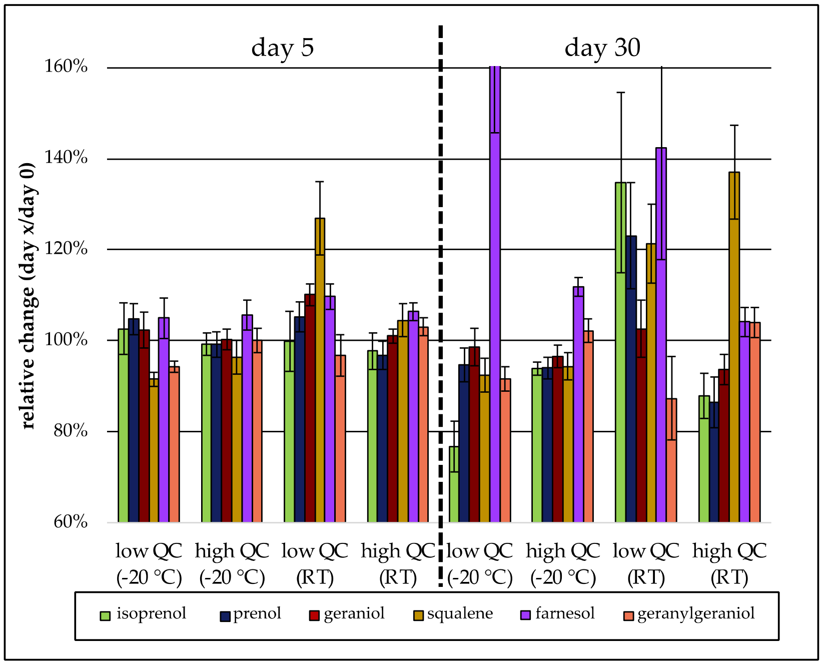

Figure S6. Analyte stability over 30 days. The relative response of every analyte was calculated as the ratio between analyte response on day 5 or 30 divided by analyte response on day 0. Left side: relative change after 5 days. Right side: relative change after 30 days. The error bars show the SD of the replicates (n=6).

S2.7 Recovery

Recovery was investigated in addition to the validation process due to the use of different matrices, that have the potential to influence the extractability of analytes. Recovery was tested using three pyrophosphates (dimethylallyl pyrophosphate, farnesyl pyrophosphate and geranylgeranyl pyrophosphate) in equimolar concentrations to low QC, medium QC and high QC samples in two matrices (*S. cerevisiae*, *A. fumigatus*). The average recovery of prenol, the alcohol originating from dimethylallyl pyrophosphate, was 107% in *S. cerevisiae* and 85% in *A. fumigatus* (**Figure S7**). The mean recovery for farnesol was 133% (*S.*

cerevisiae) and 109% (*A. fumigatus*). For geranylgeraniol the mean recovery was 44% (*S. cerevisiae*) and 37% (*A. fumigatus*). Even though recovery differed from an expected 100% value, the values showed the same tendencies for both matrices and are therefore comparable and independent of the fungal cell matrix. In addition, the SD for the individual values in *S. cerevisiae* was below 10% for all replicates besides geranylgeranyl pyrophosphate at the low QC (**Table S5**, *S. cerevisiae*). In *A. fumigatus* SD was below 10% for all analytes besides farnesyl pyrophosphate at the low QC (**Table S5**, A. *fumigatus*).

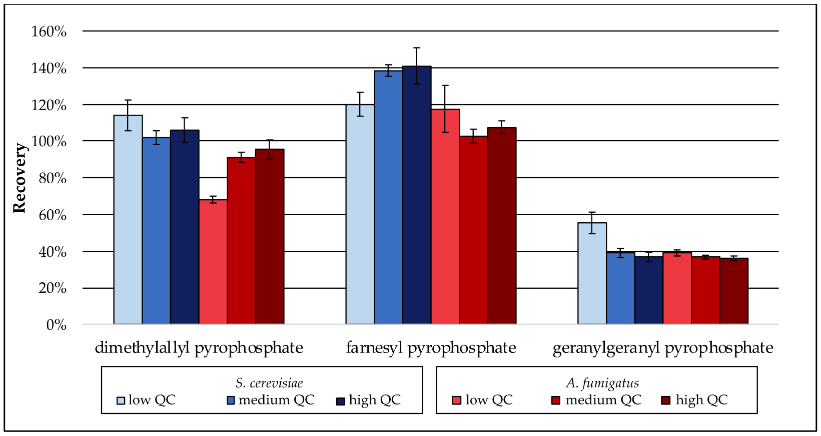

Figure S7. Recovery of three representative pyrophosphates from *S. cerevisiae* and *A. fumigatus* matrix. Error bars represent the standard deviation from six replicates.

Table S5. Relative standard deviation at three different concentrations (n=6).

	dimethylallyl pyrophosphate RSD [%]	farnesyl pyrophosphate RSD [%]	geranylgeranyl pyrophosphate RSD [%]
		S. cerevisiae	
low QC	7	5	10
medium QC	4	2	6
high QC	6	7	7
		A. fumigatus	
low QC	3	11	4
medium QC	3	4	3
high QC	6	3	4

3. References

1. Guideline on bioanalytical method validation. Available online: https://www.ema.europa.eu/en/documents/scientific-guideline/guideline-bioanalytical-method-validation_en.pdf (accessed on 28 March 2023).
